# Liver Involvement during SARS-CoV-2 Infection Is Associated with a Worse Respiratory Outcome in COVID-19 Patients

**DOI:** 10.3390/v15091904

**Published:** 2023-09-10

**Authors:** Ciro Romano, Domenico Cozzolino, Riccardo Nevola, Marianna Abitabile, Caterina Carusone, Francesca Cinone, Giovanna Cuomo, Francesco Nappo, Ausilia Sellitto, Giuseppina Rosaria Umano, Luigi Elio Adinolfi, Aldo Marrone, Luca Rinaldi

**Affiliations:** 1 COVID Center, Division of Internal Medicine, Department of Advanced Medical and Surgical Sciences, “Luigi Vanvitelli” University of Campania, 80131 Naples, Italy; riccardo.nevola@unicampania.it (R.N.); aldo.marrone@unicampania.it (A.M.); luca.rinaldi@unicampania.it (L.R.); 2 Department of Precision Medicine, “Luigi Vanvitelli” University of Campania, 80131 Naples, Italy; domenico.cozzolino@unicampania.it (D.C.); giovanna.cuomo@unicampania.it (G.C.); 3 Department of Woman & Child Health and General and Specialist Surgery, “Luigi Vanvitelli” University of Campania, 80131 Naples, Italy; giuseppinarosaria.umano@unicampania.it

**Keywords:** COVID-19, SARS-CoV-2, liver enzymes, interstitial pneumonia

## Abstract

Coronavirus disease of 2019 (COVID-19), caused by severe acute respiratory syndrome coronavirus 2 (SARS-CoV-2), may be complicated by life-threatening interstitial pneumonia. SARS-CoV-2 infection may also damage several tissues and/or organs beyond the lungs, including the liver. However, controversy still exists as to whether SARS-CoV-2-induced liver alterations can have an impact on the outcome of COVID-19. The aim of this study was therefore to assess whether SARS-CoV-2-infected patients with liver abnormalities at the time of hospital referral had a worse outcome with respect to patients with no liver biochemistry alterations. To this end, the medical records of 123 patients admitted to our COVID center between the end of 2020 and spring 2021 were retrospectively reviewed. Patients were divided into two groups: those with normal liver biochemistries (group 1, 77 patients) and those with altered liver function tests (group 2, 46 patients). Serum levels of aminotransferases (AST and ALT) and bile duct cell injury markers (γ-GT and ALP) were used to dichotomize patients. A higher percentage of patients with liver enzyme alterations were found to develop COVID-19 pneumonia with respect to group 1 patients (74% vs. 65%); moreover, they needed more days of respiratory support and, more importantly, more intensive administration of supplemental oxygen. A statistically significant correlation was also found between aminotransferase levels and duration of respiratory support. The mortality rate was not superior in group 2 vs. group 1 patients. In conclusion, liver abnormalities on admission predisposed COVID-19 patients to development of more severe interstitial pneumonia, because of a longer requirement for supplemental oxygen and a more intensive respiratory support, indicative of a worse disease evolution in these patients.

## 1. Introduction

Coronavirus disease of 2019 (COVID-19), caused by the recently identified severe acute respiratory syndrome coronavirus 2 (SARS-CoV-2), is primarily feared for interstitial pneumonia, its main life-threatening complication [1,2,3,4]. However, the clinical presentation of SARS-CoV-2 infection may be rather heterogeneous, ranging from asymptomatic or mild to severe or even fatal cases [1,2,3,4]. The main symptoms include fever, sore throat, dry cough, fatigue, myalgia, and dyspnea; moreover, anosmia and ageusia, which were frequently reported by patients during the first pandemic wave, were considered red flags for possible SARS-CoV-2 infection at that time. Other complaints, variably reported by COVID-19 patients, included headache, chest pain, hemoptysis, sputum production, rhinorrhea, nausea, diarrhea, vomiting, skin rash, cognitive impairment, and seizures [1,2,3,4]. Thus, although puzzling cases of pneumonia, clustering in some hospitals in Wuhan city, China, were the main presenting manifestation of COVID-19 at the outset of the pandemic early in December 2019 [1,2,3,4], subsequent studies have shown that SARS-CoV-2 infection may indeed damage several tissues and/or organs beyond the lungs, including the liver [5,6,7,8,9,10,11,12,13]. Accordingly, several reports have demonstrated an association between SARS-CoV-2 infection and abnormalities of liver biochemistry, such as increased aminotransferase levels and markers of bile duct cell injury [5,6,7,8,9,10,11,12,13]. Elevated liver enzymes are commonly observed during the disease course and have been suspected to be associated with a more severe and longer COVID-19 duration [7,8,9,11,13]. Thus, patients with evidence of concurrent liver involvement by SARS-CoV-2 infection should be subjected to close monitoring until COVID-19 resolution. On the other hand, patients with advanced chronic liver disease (CLD) have been shown to be at a significantly increased risk of poor outcome in the setting of COVID-19. Specifically, patients with cirrhosis and hepatocellular carcinoma (HCC) tend to have a more severe COVID-19 course as well exacerbation and progression of the liver disease, possibly resulting in liver failure [14,15]. Moreover, a condition dubbed COVID-19-associated secondary sclerosing cholangitis (SSC) has been recently described as a rare, late complication of severe COVID-19 [16,17]. This condition appears to develop almost exclusively in patients with a known underlying chronic liver disorder experiencing a severe course of COVID-19 (e.g., patients requiring admittance to and prolonged stay in intensive care facilities); moreover, patients with nonalcoholic fatty liver disease (NAFLD)/nonalcoholic steatohepatitis (NASH) and metabolic risk factors seem to be at particular risk for COVID-19-associated SSC [16,17]. This complication has been reported to have a dismal prognosis with rapid progression to advanced chronic liver disease, likely favored by a combination of factors, namely, hypoxemia, endothelial dysfunction, coagulopathy, systemic inflammatory response syndrome, extracorporeal membrane oxygenation (ECMO) and drug-induced liver injury, which synergistically come into play in critically ill patients who need long stays in intensive care units [16,17].

Whereas the reason why SARS-CoV-2 shows an elevated tropism for the airways has been recognized in the high expression of its cellular entry receptor (i.e., the angiotensin-converting enzyme [ACE] 2) in the human lower respiratory tract, the mechanism by which the same virus affects the liver remains poorly characterized, although it may result from a combination of direct virally mediated injury and the immune-mediated inflammatory response [18,19]. Indeed, ACE2 is also expressed in bile duct cells, which suggests that SARS-CoV-2 may as well infect these cells and cause the observed abnormal liver function tests in COVID-19 patients. Moreover, the presence of viral RNA in liver tissues has been confirmed by liver biopsies. Nevertheless, controversy still exists as to whether SARS-CoV-2 does induce liver alterations, whether these can have an impact on the outcome of COVID-19 or whether CLD may affect COVID-19 severity [6,7,9,20,21,22].

The aim of this study was therefore to assess whether patients with liver abnormalities at the time of SARS-CoV-2 infection had a worse outcome with respect to patients with no abnormalities in liver biochemistry, particularly with regard to its more feared complication, that is, COVID-19 pneumonia. Conversely, the impact of a known, pre-existing liver disorder (e.g., chronic hepatitis B, chronic hepatitis C, liver cirrhosis due to any cause, autoimmune hepatitis, primary biliary cholangitis, and hepatocellular carcinoma) on COVID-19 outcome was beyond the scope of this work.

## 2. Patients and Methods

### 2.1. Clinical Record Review

We retrospectively reviewed the medical records of all consecutive COVID-19-positive subjects admitted to our Internal Medicine COVID center from December 2020 to May 2021. Diagnosis of SARS-CoV-2 infection was carried out following reverse transcriptase (RT)-polymerase chain reaction (PCR) amplification of viral RNA from nasopharyngeal swab samples. All patients were preliminarily assessed in the emergency room of nearby hospitals and then immediately transferred to our COVID center (same-day admission). Initial management strategies were therefore the same for all patients. Patients with severe respiratory distress already requiring intensive treatments were not suitable for treatment in our division and were referred to the appropriate wards. Likewise, patients from nursing homes were not treated at our institution.

### 2.2. Eligibility Criteria

To be eligible for the study, patients should have undergone routine laboratory tests, evaluation of PO_2_/FiO_2_ (P/F) ratio, and chest computed tomography (CT) scan, with CT severity score (CTSS) assessment according to Chung et al. [23]. Briefly, each of the five lung lobes was accurately evaluated in order to establish the extent of inflammatory involvement, which was preliminarily categorized as none (0%), minimal (1–25%), mild (26–50%), moderate (51–75%), or severe (76–100%). Lobes with no inflammatory involvement were given a score of 0, minimal involvement corresponded to a score of 1, mild involvement was assigned a score of 2, moderate involvement had a score of 3, and severe involvement obtained a score of 4. Thereafter, an overall lung severity score was calculated by summing the five lobe scores (range of possible scores: 0–20). Chest CT scan was performed on the same day of hospital admission by a dedicated radiologist with expertise in COVID-19 pneumonia.

### 2.3. Twenty-Four Hour Telemetry, Lab Tests, Clinical Data, and Standard Treatment

All patients were monitored 24 h a day by telemetry (Dräger Vista 120 S, Drägerwerk AG, Lübeck, Germany) for respiratory and cardiovascular function throughout hospitalization. Laboratory tests used in this study included lactate dehydrogenase (LDH), neutrophil-to-lymphocyte ratio (NLR), lymphocyte-to-monocyte ratio (LMR), erythrocyte sedimentation rate (ESR), C-reactive protein (CRP), ferritin, fibrinogen, D-dimer, aspartate aminotransferase (AST), alanine aminotransferase (ALT), alkaline phosphatase (ALP), γ-glutamyltransferase (γ-GT), and albumin. Values obtained on admission at first blood draw were used for statistical analyses. Clinical data included age, gender, type of respiratory support (detailed below), time to first oxygen administration (Tt1stO_2_), i.e., the number of days between admission and need for supplementary oxygen administration, and, finally, time to ventilation weaning (TtVW), i.e., the number of days of supplementary oxygen until return to ambient air respiration. All patients were treated according to the standard treatment at the time of admission, namely, dexamethasone, low-molecular weight heparin, and supportive measures, as appropriate [24], whereas comorbidities were treated according to their specific, established guidelines. Remdesivir was considered in select patients on a compassionate basis but was never started on the first day of hospital admission.

### 2.4. Categorization of Patients with Abnormal Liver Biochemistry

Patients were divided into two groups based on the results of entry tests for AST, ALT, ALP, and/or γ-GT. Although several other parameters possibly linked to liver function, such as cholinesterase, prothrombin time, albumin, and ferritin, could likewise suggest hepatic dysfunction, they could not be strictly linked to liver damage as their alterations could also have represented the result of systemic inflammation and coagulation derangements induced by SARS-CoV-2 [1,2,3,4,25]. Therefore, they were not used to dichotomize patients because of possible interference by different pathophysiologic mechanisms. The two groups were as follows: group 1, including patients with no alterations in liver biochemistry, and group 2, consisting of patients with abnormalities in one or more liver enzyme serological tests. To avoid bias due to laboratory oscillations, i.e., inclusion of patients with liver enzyme values of 1–2 U/L over the upper limit of normal within group 2, which would have led to a potential overestimation of the patients with abnormal liver tests, only titers at least 1,1 folds over the upper limit of normal were arbitrarily considered abnormal. All patients were tested for common hepatitis viruses. None of the patients was affected by cirrhosis. None of the patients had been previously diagnosed with autoimmune hepatitis, primary biliary cholangitis, or overlap autoimmune syndromes. No history of drug abuse or prolonged use of hepatotoxic drugs was reported. Ultimately, with the interpretation limits of a retrospective study, the abnormal liver results of the enrolled patients were primarily considered to be related to SARS-CoV-2 infection.

### 2.5. Types of Respiratory Support

Respiratory support (RS) ranged from no supplemental oxygen (ambient air respiration) to orotracheal intubation (OTI). The outcome of RS was unpredictable at the time of admission, as some patients in ambient air did not require any RS throughout the hospital stay, whereas others proceeded to need RS of variable intensity. Specifically, the respiratory support was categorized as follows: (1) no RS (ambient air respiration); (2) oxygen delivered through nasal cannulas (NC); (3) oxygen delivered through high-flow nasal cannulas (HFNC); (4) oxygen delivered by means of continuous positive airway pressure (CPAP) or non-invasive ventilation (NIV); (5) need for OTI and mechanical ventilation. To simplify statistical analysis, patients were split just into two main groups: (a) those remaining in ambient air respiration plus those requiring oxygen delivered through either NC or HFNC (group 1, no or mild/moderate RS); and (b) those needing CPAP, NIV, up to OTI with mechanical ventilation (group 2, intensive RS).

#### Criteria for and Settings of Intensive Respiratory Support

Patients underwent intensive RS as soon as they developed a P/F below 200 despite delivery of a high fraction of inspired oxygen (FiO_2_) through HFNC. Initial positive end-expiratory pressure (PEEP) for CPAP was uniformly set at 7 cm H_2_O, with titration upwards or downwards according to peripheral oxygen saturation on arterial blood gas tests and respiratory rates. The delivered oxygen (FiO_2_) was titrated to warrant SpO_2_ > 92%. Low starting pressures were applied because of the risk for pneumothorax and/or pneumomediastinum [26], which have been reported as CPAP complications, particularly during the first pandemic wave, when higher pressures were routinely used being the pathophysiology of COVID-19 pneumonia much less defined. Patients on CPAP with no clinical improvement, as inferred by unimproved values of SpO_2_ and/or P/F after a 4–6 h trial, and/or developing signs of respiratory fatigue (respiratory rate > 30 breaths/min, increased serum lactate, recruitment of accessory respiratory muscles) and/or hypercapnia were switched to NIV with pressure support (pressure support ventilation modality). Initial pressure support was set at an intermediate level, i.e., 6–8 cm H_2_O, with titration upwards to ensure a tidal volume of 7–8 mL/kg, or downwards, if not tolerated. Patients with either hypoxemia unresponsive to NIV (PaO_2_ unable to reach at least 60 mmHg) or with P/F persistently < 100 despite noninvasive RS for at least 6 h or developing hemodynamic instability, underwent OTI and invasive mechanical ventilation, in absence of contraindications or patient refusal.

### 2.6. Statistical Analysis

Continuous variables were checked for normality with the Kolmogorov–Smirnov test. Differences between continuous variables were investigated using the Student *t*-test for independent samples and the Mann–Whitney U test according to distribution. Spearman correlation analysis was performed to investigate the correlation between liver enzymes, inflammatory markers, and respiratory parameters. Univariate logistic regression analyses were performed to identify candidate variables for multivariate analysis in order to estimate the risk of respiratory support. Variables showing association (*p* < 0.05) with respiratory support were included in the multivariate analysis. Chi-square tests were performed for differences in categorical variables. Continuous variables are expressed as medians (interquartile range, IQR), whereas categorical variables are expressed as frequencies. All the analyses were performed using SAS^®^ University Edition (SAS Institute Inc., Cary, NC, USA). Values of *p* < 0.05 were considered statistically significant.

## 3. Results

**Figure 1:**
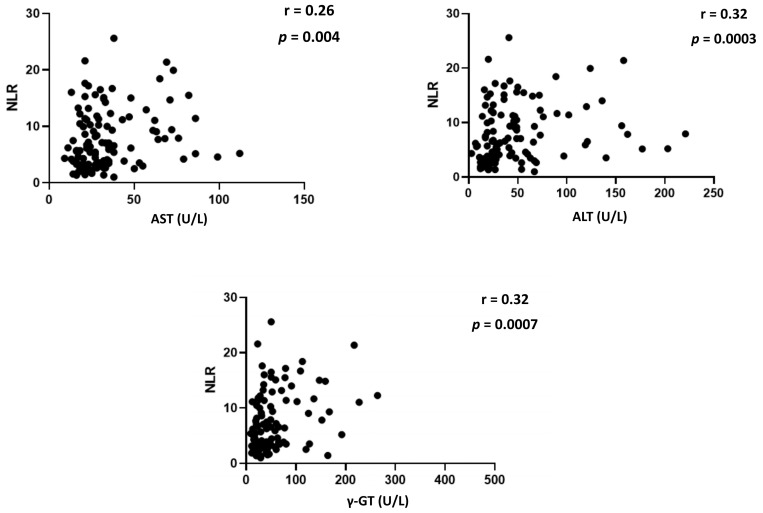
Spearman correlations between serum levels of liver enzymes (AST, ALT, γ-GT) and NLR. Abbreviations: AST, aspartate aminotransferase; ALT, alanine aminotransferase; γ-GT, γ-glutamyltransferase; NLR, neutrophil-to-lymphocyte ratio.

The two groups (group 1, including 77 patients with no liver test alterations; group 2, comprising 46 patients with altered liver biochemistries) were age- and sex-matched and no differences were observed in the number of comorbidities (Table 1). Briefly, patients with liver abnormalities were found to have a greater condition of systemic inflammation, as inferred by higher median values of ESR, CRP, and fibrinogen. Consistently, the NLR, which is considered an easy-to-obtain surrogate marker of host immunoinflammation (it is, in fact, easily calculated from a standard, relatively inexpensive full blood count) since it has been shown to correlate with clinical outcome in a variety of diseases, ranging from cardiovascular disorders to cancer and even COVID-19 itself [25,27], was found to be significantly higher in patients with liver abnormalities as compared to patients with no evidence of liver involvement. Moreover, a significant correlation was found between AST, ALT, γ-GT levels, and NLR (Figure 1).

Abnormalities in coagulation parameters (D-dimer, fibrinogen, etc.) were more frequently seen in patients with liver abnormalities, and these were essentially related to COVID-19 severity [25,28] rather than liver disease, as none of the patients had evidence of cirrhosis. The characteristics of the patients are summarized in Table 1.

### 3.1. Liver Abnormalities and Severity of Pneumonia on Patient Admission

Fifty out of 77 group 1 patients (65%) and 34 of 46 group 2 patients (74%) were admitted with or developed COVID-19 pneumonia during hospital stay. When considering CTSS and P/F to stage pneumonia severity on patient admission, a significant difference was observed between the two groups only using the radiological score (*p* = 0.01, Table 1). Albeit group 2 patients tended to show lower values, P/F was not different between the two groups, meaning that all patients had similar impairment of blood oxygen supply on admission (Table 1); however, COVID-19 pneumonia can have an unpredictable evolution even in patients with similar P/F at hospital entry [1,2,3,4]. Moreover, based on the score proposed by our group [29], group 2 patients were at risk of severe outcome because of significantly higher CTSS and lactate dehydrogenase (LDH) values (Table 1). Specifically, previous work on COVID-19 patients admitted to our COVID center identified cut-off values of >7 and >328 U/L for CTSS and LDH, respectively, as predictive of a worse evolution of COVID-19 pneumonia in SARS-CoV-2-infected subjects [29]. As shown in Table 1, the median values of CTSS and LDH in group 2 patients were indeed 10 and 335 U/L, respectively, suggesting an increased risk for severe evolution of COVID-19 pneumonia.

### 3.2. Liver Abnormalities and Respiratory Support

Whereas there were no statistically significant differences in terms of chances to undergo respiratory support (RS) between the two groups, patients with liver abnormalities were shown to need more intensive RS when compared to patients with no biochemical alterations of liver enzymes. Specifically, as detailed in the Methods section, when patients were dichotomized into two groups to facilitate statistical analysis, i.e., those requiring no or mild to moderate RS (i.e., no oxygen supplementation or oxygen delivered by either nasal cannulas or high flow nasal cannulas) and those undergoing intensive respiratory support (i.e., need for CPAP, NIV, or OTI), 50% of group 2 patients were found to have undergone intensive support, as compared to only 28.6% of group 1 patients (*p* = 0.02, Fisher’s exact test). At univariate analysis, the odds ratio for intensive respiratory support was 2.5 folds higher (95% CI 1.2–5.3) for group 2 patients as compared to group 1 patients. Multivariate analysis identified only P/F as a predictive factor for intensive respiratory support.

### 3.3. Liver Abnormalities and Length of Respiratory Support

**Figure 2:**
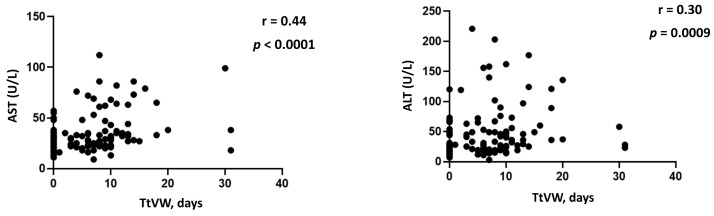
Spearman correlations between aminotransferase serum levels and duration of respiratory support. Abbreviations: AST, aspartate aminotransferase; ALT, alanine aminotransferase; TtVW, time to ventilation weaning.

Interestingly, when summing up the total days of respiratory support before resumption of ambient air respiration (TtVW), a trend to a statistically significant difference was observed between the two groups (Table 1). Specifically, group 2 patients appeared to have needed oxygen supplementation for a median longer period when compared to group 1 patients (8 vs. 5.5 days, *p* = 0.06). The smaller number of patients in group 2 as opposed to group 1 may have likely prevented reaching statistical significance. Nonetheless, a significant correlation was found between aminotransferase levels and duration of RS (Figure 2).

### 3.4. Liver Abnormalities and Mortality

No large differences were detected between the two patient groups. In fact, three patients from group 1 and only one patient from group 2 died of respiratory failure due to COVID-19 interstitial pneumonia.

### 3.5. Liver Abnormalities and Hepatitis Virus Tests

Among the 46 patients with liver abnormalities, 3 had been vaccinated against HBV, 10 showed evidence of a past HBV infection, 4 were HBV carriers with no replication (HBV-DNA negativity). The remaining patients tested negative for viral hepatitis markers, thus none of group 2 patients had evidence of HCV infection. Among the 77 patients with normal liver biochemistries, 27 had evidence of a past HBV infection, 7 had been vaccinated against HBV, 3 were positive for HCV antibodies. The remaining patients displayed negative markers for active or past viral hepatitis infections.

### 3.6. Liver Abnormalities at Discharge

Among the 46 patients with liver abnormalities at admission, only 12 (26.1%) normalized liver biochemistries at hospital discharge. We do not have longer follow-up data; thus, it is unknown whether patients normalized liver enzyme tests days or weeks after discharge. Moreover, we could not rule out a possible role for a pre-existing condition of NAFLD in favoring persistence of elevated liver biochemical tests; however, predisposing conditions to NAFLD, such as type 2 diabetes, obesity, hypertension and dyslipidemia, were homogeneously distributed between the two groups, affecting 38.96% (30 out of 77 patients) and 39.13% (18 out of 46 patients) of group 1 and 2 patients, respectively.

## 4. Discussion

The recent SARS-CoV-2 pandemic outbreak, whose origin has been likely traced to exposure to wild animals sold at wet markets in Wuhan, China [1,2,3,4], at the end of 2019, has become a major health threat to the general population and healthcare workers worldwide, with continued increases in the number of cases and deaths and unbearable burden on health systems across the globe. Since the initial pandemic outbreak, our understanding of COVID-19 has improved very rapidly, with new findings accumulating on a daily basis. SARS-CoV-2 has been first recognized as a respiratory tract pathogen, COVID-19 pneumonia being its most feared complication [1,2,3,4], but more and more reports have detailed how the virus can affect many other systems as well. Although abundant and consistent evidence has been collected thus far on lung, cardiovascular, and nervous involvement in patients affected by COVID-19 [1,2,3,4,30,31,32,33], controversial data are available about the interrelationship between SARS-CoV-2 infection and the liver [5,6,7,8,9,10,11,12,13,20,21,22]. Therefore, we aimed at contributing to this unresolved issue by sharing on our experience.

Briefly, patients with altered liver enzyme levels appeared to fare worse than patients with no evidence of liver involvement. Since COVID-19 pneumonia is the most feared complication of SARS-CoV-2 infection, the primary aim of our study was to assess whether liver involvement could impact on severity of lung inflammation. Indeed, group 2 patients tended to need respiratory support for more days than group 1 patients, and, even worse, they required a more intensive respiratory support, as judged by the modality of oxygen administration. The combination of higher CTSS scores, indicating a more widespread lung inflammation, and higher LDH serum levels, a surrogate index of lung tissue damage, observed more frequently in group 2 patients, has indeed been shown by our group to predispose to a worse respiratory outcome [29]. Patients with severe COVID-19 pneumonia are also known to display features of systemic inflammation. Accordingly, group 2 patients had significantly higher scores in several acute phase reactants and inflammation markers, including ESR, CRP, fibrinogen, ferritin, NLR. It is interesting to note that both groups showed similar P/F values on admission, which means that group 2 patients did not start the study with a more severe lung involvement than group 1 patients, rather, worsening occurred during the disease evolution.

Age and comorbidities have been shown to negatively impact on the outcome of COVID-19 [34,35]; however, there were no differences in age and the number of comorbidities between the two groups of our study. The most common comorbidities reported in patients with COVID-19 possibly linked to a worse disease outcome are hypertension, diabetes, obesity, respiratory disease, and cardiovascular disease. Our study groups were found to be homogeneously matched for these comorbidities; moreover, none of the patients in our study was suffering from acute or chronic viral hepatitis or cirrhosis. Although we could not precisely estimate the number of patients with a pre-existing condition of NAFLD on admission because of the retrospective nature of the study, its predisposing conditions, i.e., obesity, type 2 diabetes, hypertension, dyslipidemia, or their combination (metabolic syndrome), were nonetheless homogeneously distributed between the two groups. Finally, drug hepatoxicity as a cause of elevated liver enzymes was ruled out because liver biochemical tests were assayed at admission, before patients were administered the treatment for COVID-19 available at that time. In this regard, since analyzed patients were from the 2020–2021 COVID-19 wave, current drugs, including monoclonal antibodies and modern antivirals [36], able to mitigate the disease evolution in high-risk patients, were not available at that time; thus, outcome in both groups relied solely on the standard of care of that period, i.e., dexamethasone, low-molecular weight heparin, and tocilizumab. Remdesivir indeed became available as a further anti-COVID-19 drug months later patients were referred to our COVID center [37]; moreover, since tocilizumab therapy was considered only after worsening of respiratory function [38], i.e., when patients had already been classified into the high-intensity group in relation to respiratory support, this therapeutic measure did not affect the correct categorization of pneumonia severity. Current or past experimental drugs undergoing clinical evaluation for COVID-19 [36,37,38,39], including oseltamivir, lopinavir/ritonavir, ribavirin, remdesivir, and chloroquine phosphate or hydroxychloroquine sulfate, are known to be metabolized in the liver. The likelihood that the liver impairment observed in patients with COVID-19 infection could, at least in part, be due to drug hepatotoxicity may explain the considerable variation in the prevalence of liver injury observed across treatment cohorts in clinical studies. In addition, liver injury can impair metabolism, dosing, and expected concentrations of the medications, which, in turn, can increase the risk of toxicity. Conversely, our cohort may provide insights into the impact of liver abnormalities on the relatively “untouched” course of SARS-CoV-2 infection, that is, without use of the above-mentioned drugs under past or current clinical scrutiny for prevention and treatment of COVID-19.

From a medical perspective, knowing that patients with SARS-CoV-2-induced liver alterations may be at risk of a worse respiratory outcome may allow a stricter vigilance, with early recognition of worsening pneumonia and prompt notification to the intensivist personnel of their impending involvement; moreover, early recognition of worsening COVID-19 pneumonia may be lifesaving, as some patients may experience rapidly evolving respiratory distress [1,2,3,4].

Of course, our study has some limitations. The main limitations can be recognized in its retrospective design, the single center referral site, and the relatively small number of patients, which may have prevented us from identifying unrecognized bias, having a wider perspective including assessment of the role played by host genetics, as highlighted by recent studies [40], and obtaining more robust statistical results, respectively. Despite that, the strict selection of subjects with abnormal liver biochemistries and the clear relationship with the inflammatory markers as well as the intensity of the respiratory support required by the patients allowed us to confidently conclude for a possible detrimental role of liver involvement by SARS-CoV-2 on the outcome of COVID-19.

## 5. Conclusions

Liver abnormalities on admission predisposed COVID-19 patients to a worse disease evolution, as inferred by the need for a longer oxygen supplementation as well as a more intensive respiratory support, when compared to subjects with no evidence of liver involvement. The mechanisms by which liver involvement may predispose to a worse respiratory outcome are still elusive, but an interplay between the liver and the lungs may be hypothesized. For instance, liver involvement by SARS-CoV-2 may impair clearance of inflammatory mediators and alter coagulation homeostasis, which in turn may render lung involvement more severe and favor thrombosis of small vessels; hypoxemia resulting from COVID-19 pneumonia and microthrombosis may further impair liver function, thus generating a vicious cycle.

Liver abnormalities in this study appeared to be primarily associated with SARS-CoV-2 infection, since patients with pre-existing, known liver disease due to any cause were excluded from initial recruitment. However, because of the retrospective nature of the study, we cannot rule out the possibility that some of the patients could have had a pre-existing, unrecognized condition of NAFLD; nevertheless, this possible bias was lessened by the homogeneous distribution of NAFLD predisposing conditions between the two groups. Undoubtedly, patients with evidence of concurrent liver involvement by SARS-CoV-2 infection should be closely monitored until COVID-19 resolution because of possible worsening of respiratory function during hospital stay. Twenty-four-hour telemetry of respiratory and cardiovascular function is strongly advised as it may effectively assist in early recognition of vital function worsening, as per our personal experience with all SARS-CoV-2 patients admitted to our COVID center [29,30,31,34,37]. Unfortunately, the relatively small sample size likely prevented us from obtaining statistical significance for other outcome measures showing a trend toward unbalance between patients with liver impairment and patients with normal liver biochemistry tests. Prospective, multicenter studies with larger numbers of patients may likely help corroborate and strengthen these results. Moreover, to finely dissect the role of liver involvement on COVID-19 outcome, further variables, emerged from the latest studies, should be taken into account when performing statistical analyses, e.g., the host genetic predisposing background, SARS-CoV-2 variants and loads, host pre-existing immunity against the virus, biomarkers of hyperinflammation [40,41]. Nonetheless, current and upcoming more effective therapies targeting SARS-CoV-2 may help curb liver involvement during COVID-19 and lessen its impact on disease severity.

## Figures and Tables

**Table 1:** Baseline characteristics of study subjects on ward admission.

	Group 1, No Liver Involvement (*n* = 77)	Group 2, Altered Liver Enzymes (*n* = 46)	*p* Value
Age (yrs)	63 (54–73)	63 (54–69)	0.20
Sex (M, %)	61.0	71.7	0.25
AST (U/L)	25 (18–36)	49 (33–71)	**<0.0001**
ALT (U/L)	23 (20–30)	67 (33–119)	**<0.0001**
ALP (U/L)	57 (49–68)	76 (58–97)	**<0.0001**
γ-GT (U/L)	28.5 (21–41)	79.5 (60–147)	**<0.0001**
Albumin (g/dL)	3.8 (3.6–4.2)	3.7 (3.6–4.1)	0.53
C-reactive protein (mg/L)	26.7 (12.5–61.5)	44.0 (19.9–107.5)	**0.01**
ESR (mm 1st hour)	42 (29–60)	57 (37–96)	**<0.03**
Fibrinogen (mg/dl)	505 (426–646)	653 (542–829)	**0.0004**
LDH (U/L)	240.5 (206–320)	335 (263–421)	**<0.0001**
Ferritin (ng/mL)	398.5 (216–831)	958.0 (508.0–1595.0)	**<0.0001**
D-dimer (ng/mL)	242 (171–394)	303 (208–524)	**0.04**
CTSS	7 (4–10)	10 (5–13)	**0.01**
LMR	2.09 (1.27–3.00)	1.89 (1.35–2.80)	0.68
NLR	5.4 (3.1–9.9)	8.5 (4.2–13.2)	**0.02**
PO_2_/FiO_2_ (P/F)	282.4 (195.0–343.5)	249 (137–329)	0.06
Tt1stO_2_ (days)	0 (0–7)	0 (0–2)	0.17
TtVW (days) Comorbidities (no.)	5.5 (0–10.0) 2 (0–6)	8 (0–11) 2 (0–6)	0.06 0.97

Abbreviations are explained in the text. Medians and ranges (in parentheses) are reported for each variable. Bold type identifies significant values.

## Data Availability

The datasets analyzed during the current study are available from the corresponding author upon reasonable request.
